# Evaluation of deep learning-based reconstruction models on non-TOF BGO PET/CT: impact of acquisition times and BSREM penalization factors on lesion detectability and SNR

**DOI:** 10.1186/s40658-026-00919-8

**Published:** 2026-07-12

**Authors:** Anna Stenvall, David Minarik, Elin Trägårdh, Sofia Kvernby

**Affiliations:** 1 Radiation Physics, Department of Hematology, Oncology and Radiation Physics, Skåne University Hospital, 224 42 Lund, Sweden; 2 Department of Translational Medicine and Wallenberg Centre of Molecular Medicine, Lund University, Malmö, Sweden; 3 Department of Clinical Physiology and Nuclear Medicine, Skåne University Hospital, Malmö, Sweden

**Keywords:** PET/CT, Time of flight, Deep-learning, BSREM

## Abstract

**Background:**

New long field-of-view (FOV) PET scanners using bismuth germanate (BGO) detectors without time-of-flight (TOF) capability are now available. These systems incorporate deep learning-based TOF (DLb-TOF) models to compensate for the absence of TOF. There is a lack of studies systematically investigating the optimal balance between signal to noise ratio and lesion detectability across a broader range of acquisition times and β-values for these DLb-TOF models. This study aims to evaluate the trade-off between acquisition time, signal-to-noise ratio (SNR) and lesion detectability to guide optimization of clinical protocol.

**Materials and methods:**

Twenty patients referred for a clinical [^18^F]fluorodeoxyglucose (FDG) PET scan were included. Each patient received 3.5 MBq/kg of [^18^F]FDG and underwent a whole-body PET acquisition (120 s/bed) on a digital BGO PET/CT (32 cm FOV) 60 min post-injection. Data were reconstructed into images (384 × 384 matrix) representing different acquisition times (120 s, 90 s, 60 s, 45 s, 30 s and 15 s) using BSREM with β-values ranging from 50 to 1100. Three DLb-TOF models (Low, Medium, High) were applied. Volumes of interest were placed in the liver and two avid lesions per patient. SNR were calculated as SUVmean_liver_/SD_liver_ and detectability were calculated as SUVpeak_tumor_/SUVpeak_liver_.

**Results:**

SNR increased with longer acquisition times and higher β-values. DLb-TOF models improved SNR across all settings, with the Low DLb-TOF model producing the largest increase. Lesion detectability depended on the acquisition time and β-value. At longer acquisition times (120 s, 90 s), β100 provided the highest detectability, while shorter times (60–15 s) required higher β-value (β300) for optimal detectability. Among DLb-TOF models, the High model gave the best detectability overall, though the Low model performed better at lower β-values.

**Conclusion:**

SNR increased with higher β-values, longer acquisition times, and DLb-TOF application. Lesion detectability, defined as the ratio of SUV_peak_ in the lesion to SUV_peak_ in the liver, depended on the β-value, acquisition time, and the DLb-TOF model used. The Low DLb-TOF model had the best SNR but at the expense of detectability. The optimal parameters for the evaluated BGO PET/CT system, balancing SNR and lesion detectability within a clinical reasonable acquisition time, were 60–90 s with β-values of 500–300, in combination with the Medium DLb-TOF model, when 3.5 MBq/kg [^18^F]FDG was administered.

## Background

Positron emission tomography (PET) is a sensitive molecular imaging modality that has become indispensable in clinical oncology for tumor detection, staging, and therapy monitoring [1]. Beyond oncology, PET is increasingly utilized for non-oncological applications such as neurology [2] and cardiology [3], but also in the assessment of various inflammatory, infectious, and physiological processes due to its ability to provide quantitative and functional information at the molecular level [4].

A new generation of longer field of view (FOV) digital PET/CT scanners based on bismuth germanate (BGO) scintillation crystals combined with a silicon photomultiplier (SiPM) are now available [5, 6]. The BGO crystals exhibit a high linear attenuation coefficient at 511 keV, are free from intrinsic radioactivity, and are more cost-effective to manufacture compared to lutetium-based alternatives such as LSO and LYSO [7]. However, the BGO crystals are characterized by a low light yield (photons / 511 keV photon) and poor time resolution, primarily due to their prolonged scintillation decay time and thereby preventing the use of time of flight (TOF) information.

To compensate for the lack of TOF, deep learning-based (DLb) post-processing models have been developed to emulate the benefits of TOF by enhancing image quality resembling the effect of TOF on non-TOF data [8]. For the OMNI Legend PET/CT-system (GE Healthcare), three levels of deep learning-based models (Precision Deep Learning (PDL; GE Healthcare)) are available for [^18^F]fluorodeoxyglucose (FDG) data, where each level is trained to optimize performance under various noise levels and image contrast conditions relating to the effect of true TOF information [9]. These DLb-TOF models were trained exclusively on paired non‑ToF and ToF [^18^F]FDG PET-images of patients reconstructed with a block-sequential regularized expectation maximization (BSREM) (Q.Clear; GE Healthcare) [10]. The DLb-TOF models are therefore only applicable to images reconstructed with the BSREM reconstruction algorithm [9]. In contrast to conventional ordered subset expectation maximization (OSEM) algorithm, the BSREM introduces a relative difference prior that enables full voxel‑wise convergence while suppressing noise through a single global regularization parameter, β [11]. Consequently, BSREM has consistently been shown to improve standardized uptake value (SUV) convergence, quantitative accuracy, lesion detectability, and overall image quality compared with OSEM in both phantom and clinical evaluations [12–14].

These DLb-TOF models have been evaluated with BSREM reconstruction methods for a range of penalization factors β-values, focusing on signal-to-noise ratio (SNR) and the overall visual image quality [15, 16]. However, there remains a lack of studies systematically investigating the optimal balance between a high signal to noise ratio and a high lesion detectability within a reasonable acquisition time across a broader range of acquisition times and β-values. In particular, there is a need to investigate a measure of lesion detectability and how it is compromised either due to excessive image noise or due to over-smoothing.

The aim of this study was to evaluate the impact of varying acquisition times in combination with different BSREM penalization factors (β-values) on image quality and lesion detectability in [^18^F]FDG whole-body PET imaging using DLb-TOF models. By exploring a broad range of acquisition times and penalization factors in combination with DLb-TOF models, this study aims to evaluate the trade-off between acquisition time, SNR and lesion detectability to provide guidance for optimization of clinical protocol.

## Methods

### Subjects

Twenty patients referred to a routine clinical [^18^F]FDG PET/CT scan were prospectively included in the study. All patients provided written informed consent, and the study was approved by Regional Ethical Review Board (#2016/417, #2018/117, #2018/753, #2022-01302-02) and was performed in accordance with the Declaration of Helsinki. The patients were consecutively included with inclusion criteria of having at least two visually discernible [^18^F]FDG-avid lesion in the standard clinical image, with acquisition time of 90 s, reconstructed with BSREM β 400.

### Data acquisition

Each patient was intravenously injected with 3.5 MBq/kg of [^18^F]FDG. A whole-body PET acquisition was performed 60 min post-injection using a digital BGO-based PET/CT scanner (Omni Legend, GE Healthcare) with a 32 cm axial field of view. Data were acquired in list-mode with an acquisition time of 120 s per bed position, covering the skull base to mid-thigh. A low-dose CT was acquired for attenuation correction, with tube voltage of 120 kV with a noise index of 45.

### Image reconstruction

The PET data were reconstructed into images using BSREM with a 384 × 384 matrix. To simulate different acquisition durations, list-mode data were rebinned to generate reconstructions at 120 s, 90 s, 60 s, 45 s, 30 s, and 15 s per bed position. Each dataset was reconstructed using seven different BSREM penalization factors (β-values: 50, 100, 300, 500, 700, 900, and 1100) and processed with three DLb-TOF models (Low (LPDL), Medium (MPDL), and High (HPDL)). A reference image reconstructed with ordered subset expectation maximization (OSEM) using 3 iterations, 22 subsets, point spread function (PSF) and a Gaussian 3 mm post filter, based on the clinical acquisition time of 90 s was created for each patient.

### Data analysis

For quantitative evaluation of the image quality and the lesion detectability, spherical volumes of interest (VOI) were manually placed in the upper part of the right hepatic lobe (50 mm diameter) and in two [^18^F]FDG-avid lesions per patient. The same VOI configuration of three VOIs per patient was then copied to all rebinned image sets derived from the same acquisition, ensuring identical VOI-placements across all image sets per patient. The peak standardized uptake value (SUV_peak_), defined as the mean SUV (SUV_mean_) within a 1.0 ml VOI containing the highest mean SUV, was extracted from each VOI. The SUV_peak_, SUV_mean_, and the standard deviation (SD) were extracted from the liver VOI.

Signal-to-noise ratio (SNR) was calculated as the ratio of SUV_mean_ and the SD within the liver VOI, as described in Eq. (1). The lesion detectability is commonly evaluated using the contrast-to-noise ratio (CNR) which quantifies the visibility of a signal in the presence of background noise. Traditionally, the CNR metric is calculated by measuring the noise within a region of interest (ROI) placed in the neighboring background tissue. However, this approach may be problematic in anatomically heterogenous areas, where the variability of the background may affect the noise estimation. Moreover, the placement of the ROI is inherently subjective and can introduce potential bias and variability in the CNR values. To mitigate this variability, we propose an alternative method [17] that utilizes a background measurement from a more anatomically homogeneous organ, such as the liver, which is widely used as reference region in established quantitative standards [18]. This method aims to improve robustness and objectivity, particularly in the context of systematic reviews of image reconstruction techniques where lesion location may vary. In this study, the lesion detectability is defined as the ratio of SUV_peak_ in the lesion to SUV_peak_ in the liver (peak-to-peak), as described in Eq. (2).

1 $$SNR=\frac{{SUV}_{mean, liver}}{{SD}_{liver}}$$

2 $$P2P=\frac{{SUV}_{peak, lesion}}{{SUV}_{peak, liver}}$$

### Verification using an anthropomorphic phantom

To validate the findings obtained from the patient data, measurements were performed using an anthropomorphic image quality XL Thorax phantom (Data Spectrum, Durham, North Carolina, USA), equipped with a fillable sphere with a volume of 0.5 mL (Hollow Sphere Set 6™, Data Spectrum, Durham, North Carolina, USA). The phantom was prepared with a sphere-to-background ratio of 4:1 and filled with a homogeneous background activity concentration representative of typical liver FDG concentration in a clinical PET examination (4.8 kBq/ml) within the thorax compartment, further referred to as the phantom background. The phantom also included a liver insert filled with the same activity concentration as the phantom background. In addition, two lung inserts (water and polystyrene beads) containing no activity, as well as a liquid bone spine insert containing no activity, were incorporated.

Five repeated acquisitions were performed with an acquisition time of 120 s and data were acquired in list-mode. All scans were performed using one bed-position with the sphere positioned at the center location of the axial FOV of the PET-scanner. Each dataset was reconstructed and analyzed in the same way as the patient data, using matrix size of 384 × 384 voxels, the same range of acquisition times (120 s, 90 s, 60 s, 45 s, 30 s, and 15 s), BSREM penalization factors (β-values: 50, 100, 300, 500, 700, 900, and 1100), processed with and without the three DLb-TOF models. For extraction of quantitative data from the phantom measurements, a VOI was placed over the sphere at its known location. This VOI was propagated to all rebinned and reconstructed images, and the SUV_peak_ over the hot sphere was extracted, defined as the highest SUV mean within a 1.0 ml volume. To determine background SUV_peak_, a ROI was defined in the same transaxial slice as the sphere centre. Within this ROI, SUV_peak_ was determined by identifying the 1.0 ml VOI with the highest mean SUV, ensuring that the axial centre position corresponded to the centre of the sphere. These phantom measurements were used to verify the trends observed in the study population with respect to SNR (the ratio of SUV_mean_ and the SD within the background VOI) and peak-to-peak (SUV_peak_ in the lesion to SUV_peak_ in the background), although these phantom-based measurements cannot be fully translated to a clinical population, where physiological variability and motion may influence image characteristics and quantitative measurements.

## Results

### Study population

Twenty patients were included in the study. Patient characteristics are given in Table 1.

**Table 1:** Patient characteristics (mean (range)). Unit defined in square brackets

Gender [male/female]	6/14
Age [years]	72 (44–89)
Weight [kg]	67 (44–99)
Height [m]	1.68 (1.55–1.92)
BMI [kg/m2]	23.7 (16.4–37.3)
Administered activity [MBq]	234 (153–346)
Administered activity [MBq/kg]	3.48 (3.26–3.51)
Time between injection and imaging [min]	60.4 (55–72)

### Lesion information

For each subject, two [^18^F]FDG-avid lesions were included. The chosen lesions were small, average volume, measured as 41% of SUV_max_, was 1.2 ml with a range between 0.1 and 3.7 ml. Eight of the lesions were located in the pelvic region, six lesions in the abdomen, 20 lesions in the thorax region and six lesions were located in the head and neck region.

### Signal-to-noise ratio

**Fig. 1:**
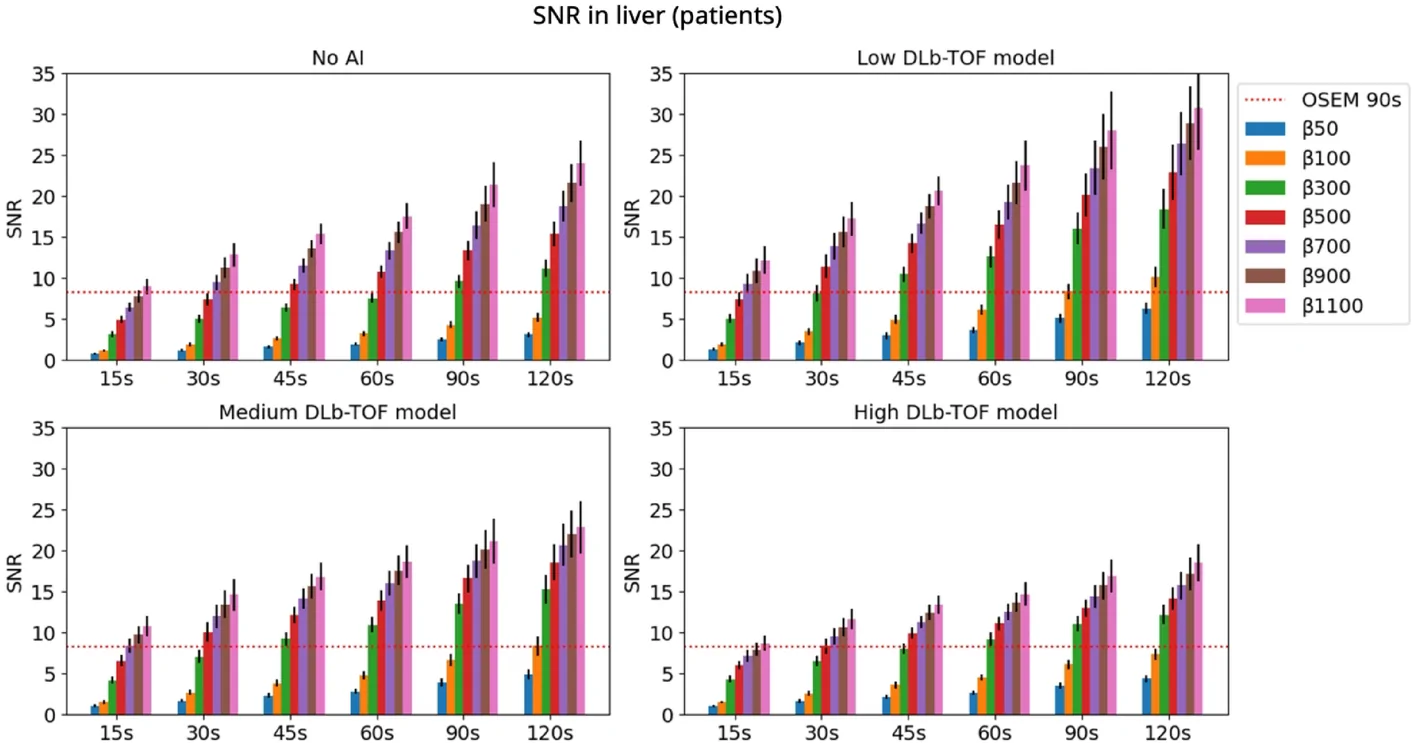
Signal-to-noise (SNR), according to Eq. (1), in lesion-free liver as a function of acquisition time and β-value without (No AI) and with the three levels of DLb-TOF model (Low, Medium and High). The dotted line represents SNR for 90 s acquisition time reconstructed with OSEM. Error bars indicate ± 1 SD

Figure 1 shows the average SNR in lesion-free liver for the 20 patients as a function of acquisition time and beta value without (No AI) and with the three levels of DLb-TOF (Low, Medium and High). The SNR demonstrated a consistent increase with both prolonged acquisition times and higher β-value, ranging from 0.7 for β50 at 15 s to 24.3 for β1100 at 120 s. For each evaluated acquisition time, the SNR increased with increasing beta value and for each β-value, the SNR increased with increasing acquisition time. This is seen for all reconstructions, both with and without deep learning-based time-of-flight. The application of DLb-TOF reconstruction models showed an increase in SNR for Low and Medium DLb-TOF model, whereas High DLb-TOF model showed an increase in SNR for beta values of 50–500 and a decrease in SNR for beta-values of 700–1100 (− 23% for 120 s acquisition time for β1100). Notably, the Low DLb-TOF model yielded the most substantial improvement, with an SNR increase of up to 104% (120 s acquisition time for β50) compared to conventional reconstruction methods. Application of High DLb-TOF model results in a less notable increase in SNR as a function of β-value for all acquisition times. Compared with the SNR obtained for the OSEM reconstruction (at acquisition time of 90 s), the similar SNR can be reached for all chosen BSREM-reconstructions, regardless of DLb-TOF model, by changing the acquisition time.

**Fig. 2:**
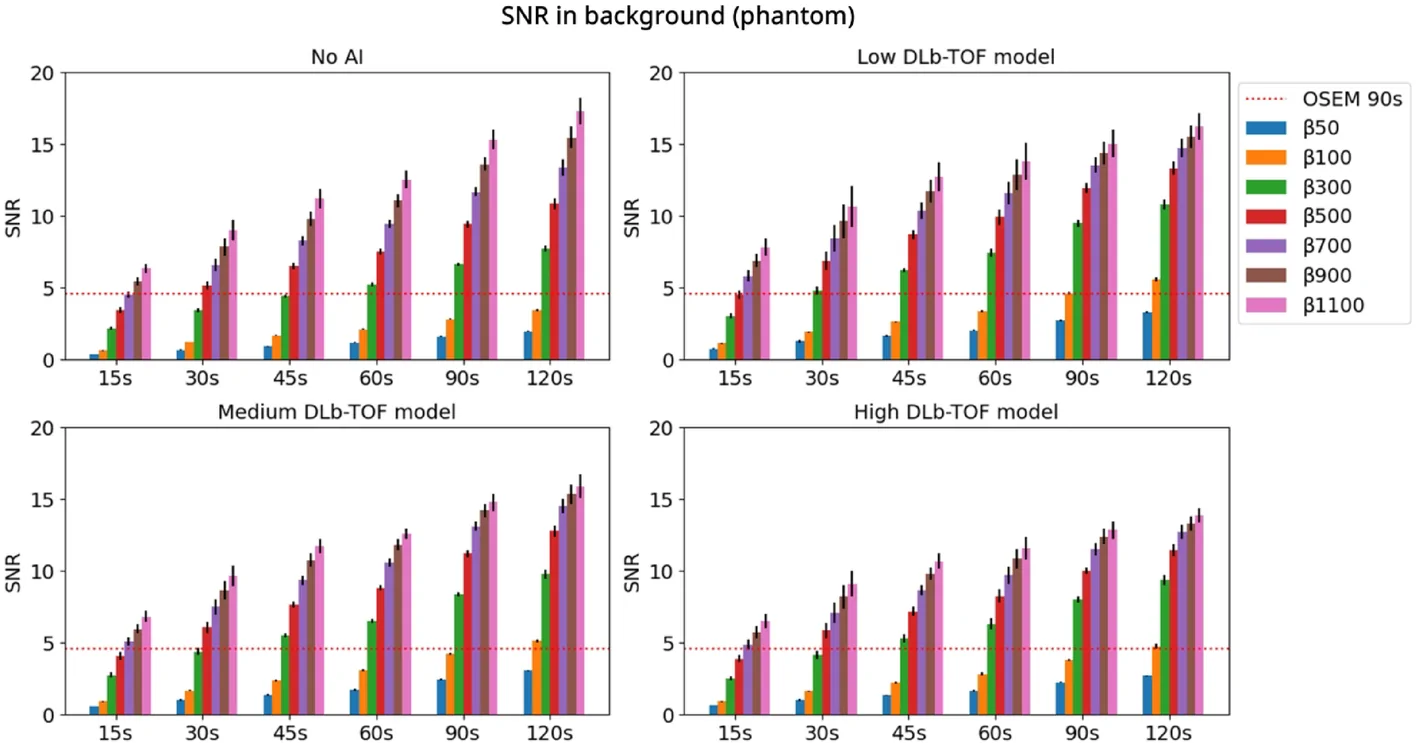
Signal-to-noise (SNR) from a homogeneous background in an anthropomorphic phantom, as a function of acquisition time and β-value without (No AI) and with the three levels of DLb-TOF model (Low, Medium and High). The dotted line represents SNR for 90 s acquisition time reconstructed with OSEM. Error bars indicate ± 1 SD

Verification of the SNR using an anthropomorphic phantom with a homogeneous background demonstrated results consistent with those observed in the patient cohort. The results are presented in Fig. 2.

### Lesion detectability

**Fig. 3:**
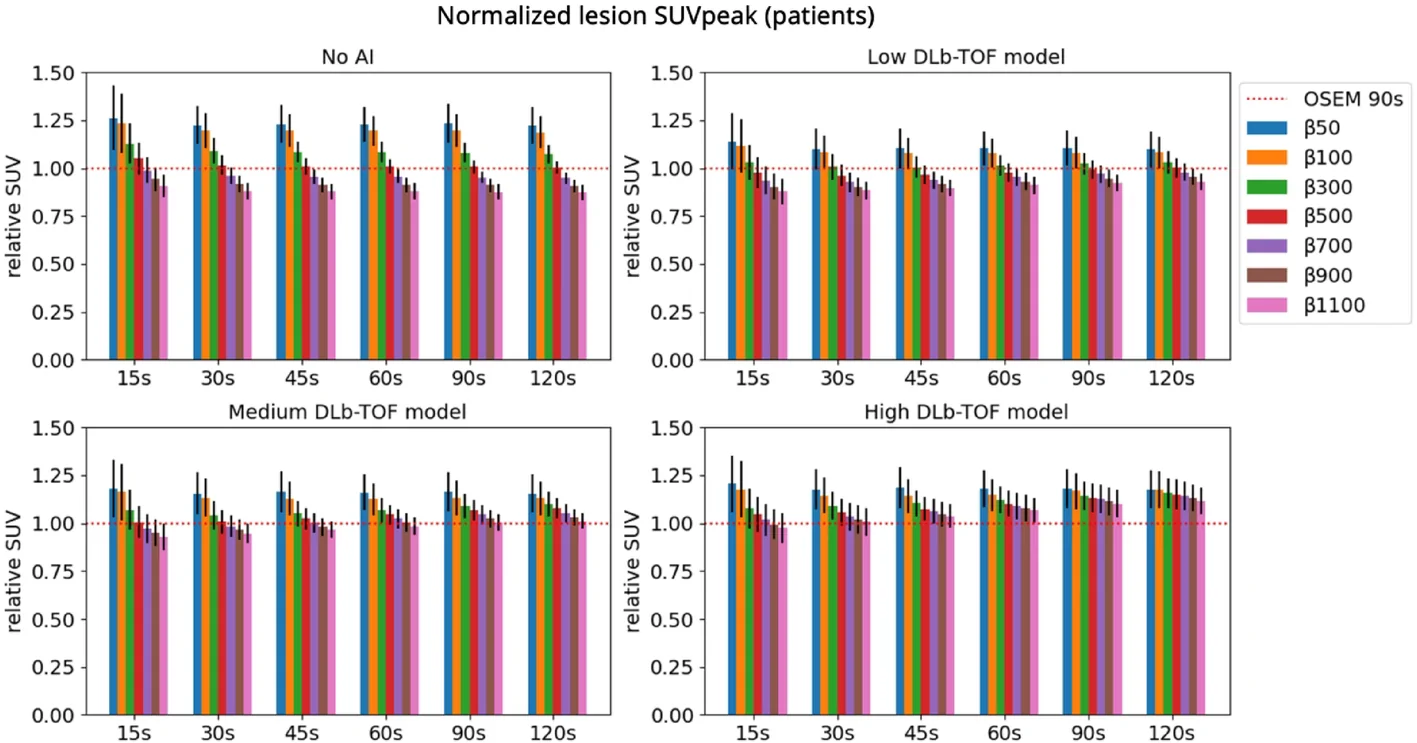
Lesion SUV_peak_ value normalized to SUV_peak_ of OSEM 90 s, as a function of acquisition time and β-value without (No AI) and with the three levels of DLb-TOF model (Low, Medium and High). The dotted line represents normalized SUV_peak_ for 90 s acquisition time reconstructed with OSEM. Error bars indicate ± 1 SD

Figure 3 illustrates that acquisition time did not affect SUV_peak_ in lesions. The SUV_peak_ in lesions consistently decreased with increasing β-values across all reconstructions, both without and with Low and Medium DLb-TOF model. However, for acquisition times of 60–120 s using the High DLb-TOF model, SUV_peak_ in lesions remained stable within the β-value range of 500–1100. All DLb-TOF models showed a smaller effect of β-value on SUV_peak_ than reconstructions with no DLb-TOF model. SUV_peak_ in lesions was highest across β-values for High DLb-TOF model compared to SUV_peak_ for the OSEM reconstruction.

**Fig. 4:**
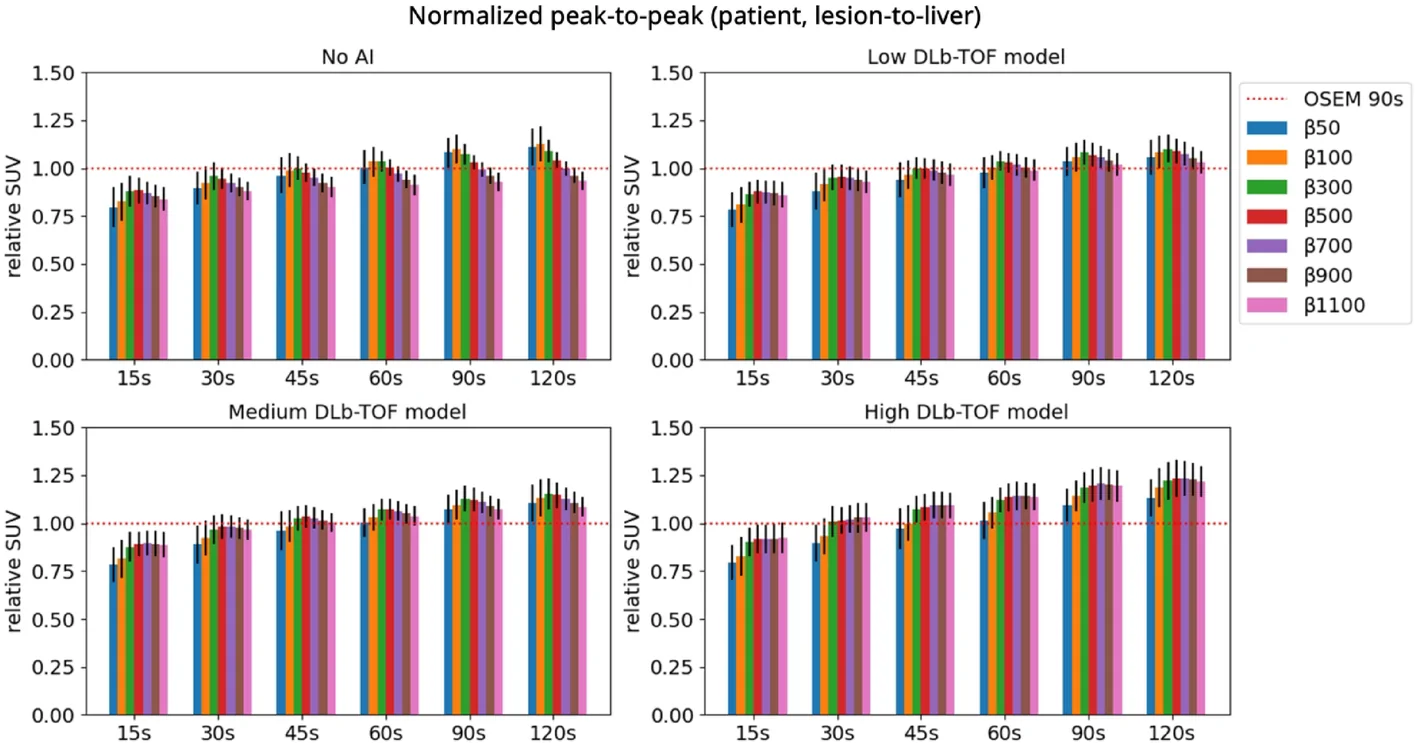
SUV_peak_ in lesion divided by patient specific SUV_peak_ in lesion free liver (peak-to-peak), according to Eq. (2), normalized to OSEM 90 s, as a function of acquisition time and β-value without (No AI) and with the three levels of DLb-TOF model (Low, Medium and High). The dotted line represents normalized peak-to-peak for 90 s acquisition time reconstructed with OSEM. Error bars indicate ± 1 SD

Lesion detectability (peak-to-peak, defined as the ratio of SUV_peak_ in the lesion to SUV_peak_ in the liver (Eq. 2) normalized to 90 s OSEM reconstruction, illustrated in Fig. 4) was found to be influenced by both acquisition time and the choice of β-value. For longer acquisition durations (90 s and 120 s), using no DLb-TOF model, optimal peak-to-peak was achieved at β100. In contrast, for shorter acquisition times (ranging from 60 to 15 s), higher β-values (β300) were necessary to maintain adequate lesion conspicuity. When evaluating the performance of DLb-TOF models, the High DLb-TOF model generally provided the highest peak-to-peak across most conditions. However, its performance varied depending on the specific combination of acquisition time and β-value. The highest peak-to-peak was seen for the longest acquisition times (90 s and 120 s), using β900. The Low and Medium DLb-TOF model achieved highest peak-to-peak at lower β-values (β300–β500) compared to the High DLb-TOF model, where highest peak-to-peak was achieved with β500 for lower acquisition times (15–45 s) and β300 for longer acquisition times. Compared to the peak-to-peak of OSEM reconstruction at 90 s, all tested reconstructions using the DLb-TOF model demonstrated higher peak-to-peak for acquisition times of ≥ 90 s with Low DLb-TOF model, and ≥ 60 s with Medium and High DLb-TOF model. However, exceptions were observed for shorter acquisition times across the different DLb-models: Low DLb-TOF model at 60 s with β300 and β500, Medium DLb-TOF model at 45 s with β300, β500, and β700, and High DLb-TOF model at 30 s with β ≥ 300 all achieved higher peak-to-peak than OSEM at 90 s.

**Fig. 5:**
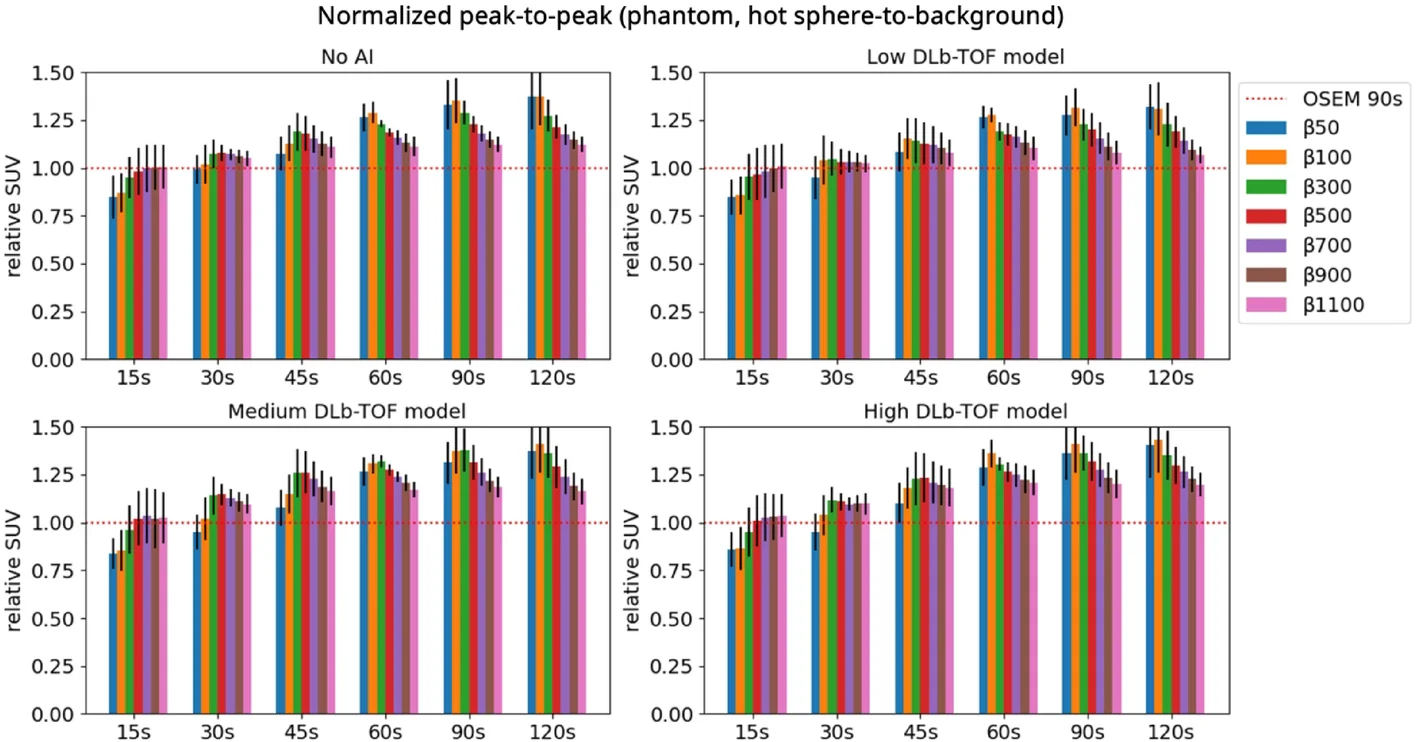
SUV_peak_ in lesion divided by SUV_peak_ in the background (peak-to-peak) in an anthropomorphic phantom, normalized to OSEM 90 s, as a function of acquisition time and β-value without (No AI) and with the three levels of DLb-TOF model (Low, Medium and High). The dotted line represents normalized peak-to-peak for 90 s acquisition time reconstructed with OSEM. Error bars indicate ± 1 SD

**Fig. 6:**
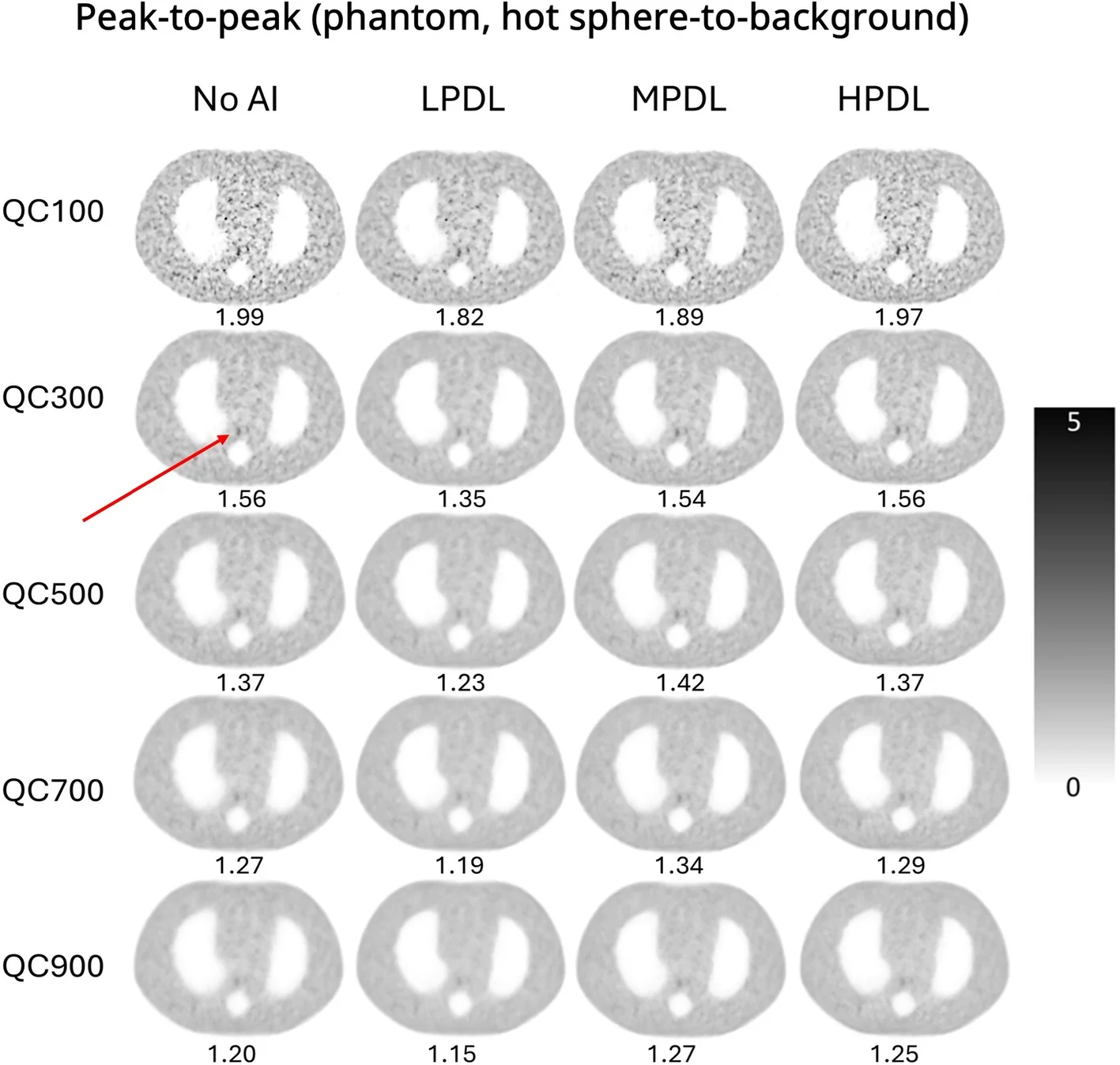
Illustration of transversal PET images showing the absolute value of peak-to-peak (SUV_peak, lesion_/SUV_peak,_ _background_) in the anthropomorphic phantom from a 45 s acquisition. Images were reconstructed using β-value 100, 300, 500, 700 and 900 using no, Low, Medium and High level of the DLb-TOF model (no AI, LPDL, MPDL and HPDL). Red arrow indicates the lesion that is represented with the numerical peak-to-peak value in the figure. Color bar indicating SUV_bw_

The peak-to-peak value measured in the anthropomorphic phantom, demonstrated results consistent with those observed for the patient population. Figure 5 presents the peak-to-peak value, normalized to 90 s OSEM reconstruction. Figure 6 shows an example of the anthropomorphic phantom, illustrating the trade-off between high SNR and reduced peak-to-peak variation.

**Fig. 7:**
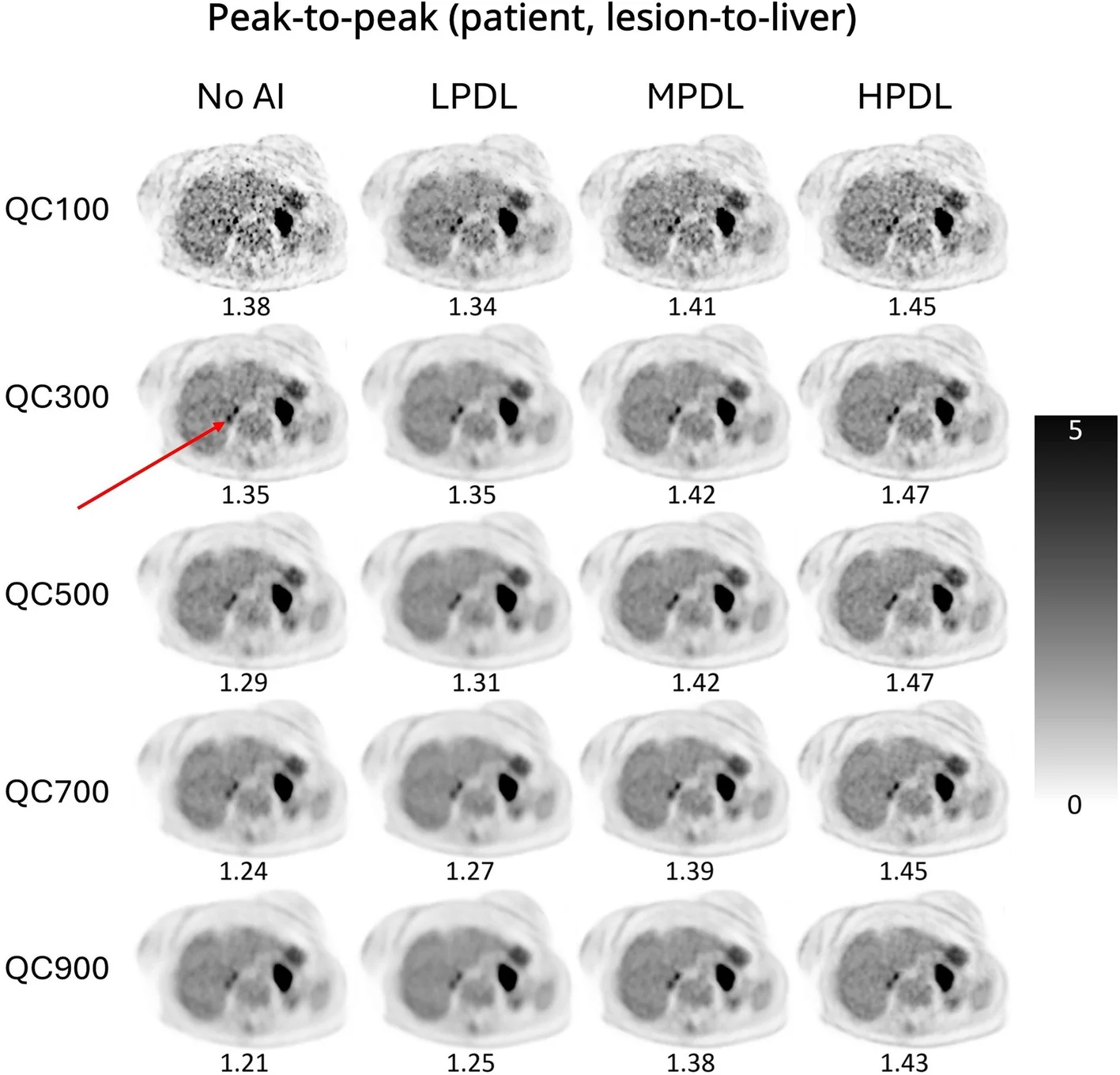
Illustration of transversal PET images showing the absolute value of peak-to-peak (SUV_peak, tumor_/SUV_peak, liver_) of an oncology patient from the 90 s acquisition. Images were reconstructed using β-value 100, 300, 500, 700 and 900 using no, Low, Medium and High level of the DLb-TOF model (no AI, LPDL, MPDL and HPDL). Red arrow indicates the lesion that is represented with the numerical peak-to-peak value in the figure. Color bar indicating SUV_bw_

**Fig. 8:**
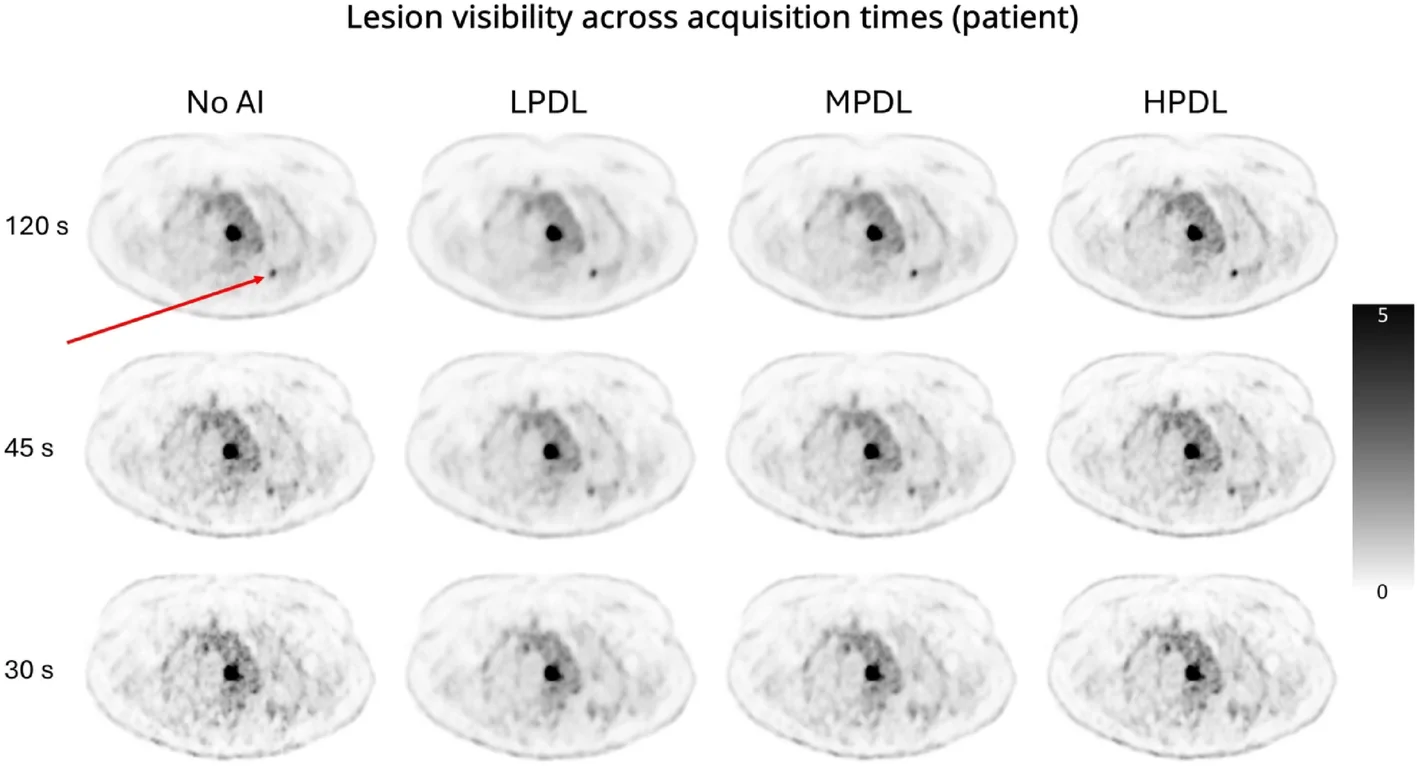
Illustration of transversal PET images showing an oncology patient from 120 s (top row), 45 s (middle row) and 30 s (bottom row) acquisition times, demonstrating a lesion that is not visible in the 30 s image. Images were reconstructed using β-value 300 using no, Low, Medium and High level of the DLb-TOF model (no AI, LPDL, MPDL and HPDL). Color bar indicating SUV_bw_

Figure 7 illustrates the absolute peak-to-peak in PET images of an oncology patient from the 90 s acquisition, showing that an increase of β-value reduces peak-to-peak, whereas an increase of the strength of the DLb-TOF model enhances it. The maximum peak-to-peak for this lesion is reached using High DLb-TOF model with β-value of 300 or 500. Figure 8, in contrast, presents a case where an acquisition time of 30 s is insufficient for the lesion to become discernible, regardless of whether DLb-TOF is applied or of its strength.

## Discussion

A new generation of longer FOV PET/CT scanners are emerging, some of which utilize non-TOF BGO detectors. In this study, we evaluated the impact of varying acquisition times and BSREM penalization factors (β-values) on lesion detectability and SNR in [^18^F]FDG whole-body PET imaging, using deep learning based-TOF (DLb-TOF) models. By systematically exploring a broad range of acquisition times and β-values in combination with DLb-TOF models, this work identifies trade-offs between acquisition time, SNR and lesion detectability, presented here as the ratio between lesion SUV_peak_ and liver SUV_peak_, to provide guidance for clinical protocol optimization. This study found that the optimal parameters for the evaluated BGO PET/CT system, balancing SNR and lesion detectability within a clinical reasonable acquisition time, were 60–90 s with β-values of 300–500, in combination with the Medium DLb-TOF model.

The SNR is a key metric of image quality, where the SNR typically increases with both acquisition time and β-value. However, higher SNR does not always equate to improved image quality and lesion detectability, as excessive noise suppression can obscure small or low-contrast lesions. In our results, SNR increased with both acquisition time and β-value, consistent with previous findings [12, 13], also when a DLb-TOF model was applied to the BSREM reconstruction, consistent with previous finding [15]. Application of DLb-TOF models to both the study population and also verified in an anthropomorphic phantom demonstrated improved SNR at the Low and Medium model levels, whereas for the High level of DLb-TOF model, a slight decrease in SNR was observed for the higher β-values. This suggests that the SNR gain from increasing β-values is attenuated when using High-level DLb-TOF models, resulting in a flatter SNR curve across β-values at fixed acquisition times (Fig. 1). In a recent reader study incorporating synthetically inserted lesions, Maronnier et al. [19] demonstrated improved noise characteristics for images reconstructed using a DLb‑TOF model in combination with BSREM, compared with BSREM without DLb‑TOF. Furthermore, it is noteworthy that a high initial SNR does not correspond to a proportionally large increase in SNR following the application of DLb-TOF model, indicating a smaller SNR enhancement at higher baseline SNR levels.

Tumor SUV_peak_ decreased with increasing β-value, likely due to noise suppression effects. However, SUV_peak_ remained stable across β-values when acquisition time increased. Interestingly, for the high-level DLb-TOF model, a slight increase in SUV_peak_ was observed at higher β-values with longer acquisition times, where SUV_peak_ at 120 s acquisition time seems to be independent of β-value (Fig. 2). Although lesion uptake was assessed using SUV_peak_ in the present study, our observed trends are consistent with previous work reporting increased lesion SUV_max_ for BSREM compared with OSEM, with a further increase when DL‑based TOF models were applied [8, 15].

Lesion detectability was assessed using the ratio of SUV_peak_ in the lesion and the SUV_peak_ in the liver (peak-to-peak), verified using the ratio of SUV_peak_ in a phantom hot sphere to SUV_peak_ in a known homogenous background. SUV_peak_ was selected over SUV_max_ since it is a more robust metric, SUV_max_ represents the single highest-intensity voxel within a defined VOI and is therefore highly susceptible to image noise while SUV_peak_ provides a more stable and reliable metric [20]. The peak-to-peak metric was chosen in preference to the commonly used CNR metric for assessing lesion detectability. A limitation with the CNR approach is that the noise is typically estimated from a ROI in the neighboring background tissue, which introduces subjectivity and potential variability in the measurement. In contrast, using the liver as a reference organ in the peak-to-peak metric provides a more anatomically consistent background across patients, although the liver does not exhibit complete homogeneous FDG distribution. Given that lesions in this study arise from different anatomical regions, this approach was considered to support a more standardized comparison across datasets. To address possible bias related to liver heterogeneity, the peak-to-peak results were verified in a known homogeneous background in an anthropomorphic phantom, where consistent trends were observed. As the liver SUV_peak_ is influenced by noise properties that vary with acquisition time and reconstruction settings, the lesion-to-liver SUV_peak_ ratio is inherently coupled to these parameters. The objectivity and reliability are particularly important in the context of systematic image reconstruction comparisons, as performed in this study.

By evaluating the SUV_peak_ ratio between the [^18^F]FDG avid lesion and the liver, the metric is intended to approximate lesion conspicuity relative to background variability in a clinically relevant reference tissue. However, without validation against observer performance, changes in the applied metrics should not be interpreted as direct evidence of improved lesion detectability but rather as indicative of changes in the relationship between lesion contrast and background noise in the liver.

The phantom experiments provide controlled support for the observed trends in these quantitative measures under standardized conditions. Nevertheless they do not fully capture the complexity of clinical imaging and thus clinical lesion detectability. Factors such as lesion heterogeneity, physiological uptake variability, and reader-dependent interpretation are not fully represented in the phantom setup and the extraction of quantitative measurements from distinct VOIs. This discrepancy highlights the gap between quantitative metrics and human observer perception. Therefore, further validation with clinical reader studies is warranted, to establish the extent to which changes in SUV_peak_ ​ ratios correspond to meaningful improvements in lesion detectability and diagnostic performance.

The lesion-to-liver SUV_peak_ ratio was influenced by both acquisition time and the choice of β-value. For longer acquisition times, 90 and 120 s, optimal peak-to-peak ratio between lesion and liver was achieved at β100, suggesting that moderate regularization of the image noise is sufficient when count statistics are high. Conversely, at shorter acquisition times, ranging from 15 to 60 s, higher β-values (β300) were required to maintain adequate lesion detectability, likely due to increased image noise at lower count statistics.

Changes in SUV_peak_ ratio between the lesion and the liver across acquisition times seem to be driven primarily by changes in liver noise, as SUV_peak_ in the lesion remained stable when changing the acquisition time. Nevertheless, due to the larger image noise, the deviation in SUV_peak_ is larger for short acquisition times and lower β-values (Fig. 3).

When evaluating the performance of DLb-TOF models, the High DLb-TOF model generally provided the highest lesion detectability across most conditions. However, its performance varied depending on the specific combination of acquisition time and β-value. Notably, the Low DLb-TOF model achieved the highest detectability at lower β-values compared with the High DLb-TOF model that achieved the highest lesion detectability at a higher beta value, indicating that the optimal β-value differs between the models. It is also noteworthy that the application of Medium and High DLb-TOF models maintained high lesion detectability across both high and low β-values, in consistence with the findings from Dadgar et al. [16]. As written above, this behavior contrasts with reconstructions without AI enhancement and with the Low DLb-TOF model, where detectability decreased at higher β-values (Fig. 4). These findings highlight the nuanced interplay between reconstruction parameters and model selection in optimizing both image quality and diagnostic performance. To further assess the absolute detectability and the benefits of DLb-TOF models compared to the absence of TOF, a dedicated phantom study is warranted.

The three levels of DLb-TOF models were trained with target TOF-images that were obtained with various β-values. The Low-level model was trained to emulate high β-value images, prioritizing noise suppression over contrast, while the High-level model targeted low β-value images, emphasizing contrast at the expense of noise [9]. Our findings reflect these training objectives: the Low-level model’s contrast can be enhanced by pairing it with a lower β-value, while the High-level model benefits from slightly higher β-values. This demonstrates two distinct pathways to achieving similar image quality.

When considering both SNR and lesion detectability, multiple combinations of acquisition time, β-value and DLb-TOF model yielded comparable SNR and detectability. Therefore, clinical decisions regarding imaging and reconstruction parameters should balance acquisition efficiency with diagnostic confidence. The optimal DLb-TOF model depends on the specific acquisition and reconstruction settings. Building on this DLb-TOF model, Mehranian et al. suggested an extension of the DLb-TOF models to additional tracers, including [^18^F]-PSMA, [^68^Ga]Ga-PSMA, and [^68^Ga]Ga-DOTATATE, aiming to improve generalizability across diverse clinical applications and tracers [21]. Hence, before such models are widely adopted for multiple tracers, it is essential to thoroughly evaluate their performance for [^18^F]FDG.

Finally, it is important to emphasize that the raw data signal is fundamental—no reconstruction algorithm or AI model can recover lesions that are not present in the original data (Fig. 8).

## Limitations

This study has some limitations. The results presented are based on an injected activity of 3.5 MBq/kg [^18^F]FDG, and for other dosages the acquisition time-activity product will change and thus also the results will be affected. This study included predominantly lean patients and patients with at least two FDG avid lesions, which may affect the generalizability of the results to more challenging clinical cases. The differences seen in reconstruction performance and acquisition times could vary and the impact of DLb-TOF models may also differ depending on patient size. The results from this study are based solely on quantitative image quality metrics, and the proposed peak-to-peak metric should therefore be considered a surrogate measure of lesion detectability. Correlation with visual assessment of image quality and lesion detectability was not addressed but represents an important topic for future investigation.

## Conclusion

SNR increased with higher β-values, longer acquisition times, and the application of DLb-TOF models. Lesion detectability, defined as the ratio of SUV_peak_ in the lesion to SUV_peak_ in the liver, depended on the β-value, acquisition time, and the DLb-TOF model used. Application of DLb-TOF models can not recover lesions that are not present in the original data. The Low DLb-TOF model had the best SNR but at the expense of detectability. The optimal parameters for the evaluated BGO PET/CT system, balancing SNR and lesion detectability within a clinical reasonable acquisition time, were 60–90 s with β-values of 300–500, in combination with the Medium DLb-TOF model, when 3.5 MBq/kg [^18^F]FDG was administered.

## Data Availability

The datasets generated during the current study are available from the corresponding author on reasonable request.

## Abbreviations

BGO: Bismuth germanate
BSREM: Block-sequential regularized expectation maximization
CNR: Contrast to noise ratio
CT: Computed tomography
DLb-TOF: Deep learning based time of flight
FDG: Fluorodeoxyglucose
FOV: Field of view
LSO: Lutetium oxyorthosilicate
LYSO: Lutetium yttrium oxyorthosilicate
OSEM: Ordered subset expectation maximization
PET: Positron emission tomography
PSF: Point spread function
ROI: Region of interest
SD: Standard deviation
SiPM: Silicon photomultiplier
SNR: Signal to noise ratio
SUV: Standardized uptake value
TOF: Time of flight
VOI: Volume of interest
