# Supplementary material for: Evaluation of deep learning-based reconstruction models on non-TOF BGO PET/CT: impact of acquisition times and BSREM penalization factors on lesion detectability and SNR
Source: EJNMMI Phys. 2026 Jul 12;13:74. doi: 10.1186/s40658-026-00919-8 (PMC13369060; doi:10.1186/s40658-026-00919-8)
Supplement: Supplementary file 1 — Supplementary Material 1 [file 40658_2026_919_MOESM1_ESM.pdf]

Sofia Kvernby
Department of Translational Medicine
Lund University
Sweden

16 September 2025

Dear Editors of EJNMMI Physics,

I am pleased to submit our manuscript entitled **"Evaluation of deep learning-based reconstruction models on non-TOF BGO PET/CT: Impact of acquisition times and BSREM penalization factors on lesion detectability and SNR"** as an original research article for consideration in EJNMMI Physics.

This study addresses a clinically relevant challenge in PET imaging: optimizing image quality and lesion detectability in the context of emerging long field-of-view PET scanners based on bismuth germanate (BGO) detectors, which lack time-of-flight (TOF) capability.

By systematically evaluating a broad range of acquisition times (15 s - 120 s), penalization factors (β-values range: β 50 - β 1100) in the image reconstruction, and deep learning-based TOF (DLb-TOF) models, we provide insights into the trade-offs between signal-to-noise ratio (SNR) and lesion detectability. Our findings suggest that a combination of 60–90 seconds acquisition time, β-values between 300–500, and the Medium DLb-TOF model yields optimal clinical performance when 3.5 MBq/kg [18F]FDG is administered.

To our knowledge, there is a lack of studies systematically investigating the optimal balance between a high signal to noise ratio and a high lesion detectability within a reasonable acquisition time across a broader range of acquisition times and β-values. In particular, there is a need to investigate a measure of lesion detectability and how it is compromised either due to excessive image noise or due to over-smoothing. The results have direct implications for protocol optimization and may contribute to improved diagnostic accuracy and patient throughput in clinical PET imaging.

We believe this work aligns well with the scope of EJNMMI Physics, particularly in quantitative imaging methodologies and promoting the integration of artificial intelligence in nuclear medicine. All authors have approved the manuscript and there is no conflicts of interest to declare.

We confirm that this work is original, has not been published previously, and is not under consideration for publication elsewhere. Part of this work will be orally presented at the EANM 2025 congress in Barcelona.

Thank you for considering our manuscript for publication. We look forward to the opportunity to contribute to your journal.

Sincerely,

Sofia Kvernby
