# Supplementary material for: Evaluation of deep learning-based reconstruction models on non-TOF BGO PET/CT: impact of acquisition times and BSREM penalization factors on lesion detectability and SNR
Source: EJNMMI Phys. 2026 Jul 12;13:74. doi: 10.1186/s40658-026-00919-8 (PMC13369060; doi:10.1186/s40658-026-00919-8)
Supplement: Supplementary file 2 — Supplementary Material 2 [file 40658_2026_919_MOESM2_ESM.docx]

Dear Editor,

Thank you for considering our manuscript EJPH-D-25-00225R1 “Evaluation *of deep learning-based reconstruction models on non-TOF BGO PET/CT: Impact of acquisition times and BSREM penalization factors on lesion detectability and SNR”* for publication in EJNMMI Physics. Please find below a point-by-point response to the reviewer’s comments. An updated manuscript with changes highlighted in yellow is also resubmitted.

**Reviewer #1:**

**Comment 1: It may be favorable to change the words, "neuro" and "cardiac" to "neurology" and "cardiology" in the introduction.**

We have changed the words “neuro” and “cardiac” according to the reviewer’s suggestion.

**Comment 2: It seems more helpful to provide the titles of figures briefly summarizing the contents of figures**

We thank the reviewer for this helpful suggestion. The titles of all figures (including graphs) have been revised to more clearly and concisely summarize the main content of each plot and figure.

**Comment 3: It may be recommended that the Lack of visual assessment be more rephrased because the Lesion detectability was tested by only quantitative measurements.**

Thank you for this important remark. In the revised manuscript, we have clarified that lesion detectability was not assessed through visual (observer-based) evaluation, but rather through quantitative metrics only. This has been rephrased in the discussion section on page 13.

**Comment 4: It may be favorable to add more detailed pictures and data of an anthropomorphic study.**

We thank the reviewer for this suggestion. We have expanded the description of the preparation, image acquisition, and analysis of the anthropomorphic phantom experiment to provide a more detailed account of this part of the study. This includes a clearer description of the phantom configuration, scan procedure, VOI placement, and how SNR and peak-to-peak metrics were derived and evaluated.

These additions aim to improve transparency and demonstrate how the phantom measurements support and contextualize the findings observed in the patient data.

**Comment 5: In overall, it may be still recommended to try more English stylistic improvement in several areas of manuscript such as "poor time resolution due to the long decay time after each detected photon and therefore lack the 13 ability to use time of flight (TOF) information" in the page 4, "~ shown for both the study population and verified using an anthropomorphic phantom, ~" and "In a recent reader study including inserted synthetic lesions, ~" in the page 11 of Discussion section.**

Thank you for this helpful suggestion. We have carefully revised the manuscript to improve the English style and clarity in the sections indicated by the reviewer. We believe these revisions have improved the flow and clarity of the manuscript.

**Reviewer #2:**

**Comment 1: The use of the liver as a reference remains a conceptual limitation. While the authors justify its use and provide phantom validation, the metric used (lesion-to-liver SUVpeak) is inherently influenced by liver noise, which itself depends on acquisition time. This creates a coupling between the optimization variable and the evaluation metric. This point is briefly mentioned in the discussion, but it would benefit from a clearer and more explicit acknowledgment.**

We thank the reviewer for this important comment. We acknowledge that the lesion-to-liver SUV_peak_ ratio represents a surrogate metric and that the background SUV_peak_, even when derived from a 1 cm³ VOI, is partly influenced by noise, does not represent a purely physiological quantity and hence cannot be regarded as a strictly defined physical measurand.

Consequently, the metric is inherently coupled to acquisition time and reconstruction parameters, as changes in liver noise directly affect the denominator. In this context, the peak-to-peak ratio should be interpreted as a contrast-related surrogate reflecting the relationship between lesion uptake and noise-affected background, rather than as an independent measure of detectability.

We have made this dependency and interpretation more explicit in the Discussion by adding a section to the Discussion (page 13).

**Comment 2: Although the added phantom experiments support the observed trends, they do not fully validate lesion detectability in a clinical sense. The manuscript now appropriately recognizes that the proposed metric is a surrogate; however, this limitation should be more clearly emphasized when interpreting the results.**

We thank the reviewer for this important comment. We agree that the phantom experiments, while supportive of the observed trends, do not fully validate lesion detectability in a clinical context.

To address this limitation, we have revised the Discussion section to more clearly emphasize that the proposed metric represents a surrogate measure rather than a direct assessment of clinical detectability. We have also highlighted that factors influencing clinical lesion detection, such as biological variability and reader interpretation, are not fully captured in the phantom experiments, and that further validation through clinical reader studies is warranted.

**Comment 3: The absence of any visual assessment remains a limitation, particularly given that the study focuses on lesion detectability. This is now mentioned, but its impact on the interpretation of the findings could be stated more explicitly.**

We agree that the absence of visual or observer-based assessment represents a limitation, particularly in the context of lesion detectability.

We have revised the manuscript to more explicitly state the implications of this limitation. Specifically, we now clarify in the Discussion section that changes in the applied quantitative metrics should not be interpreted as direct evidence of improved lesion detectability, but rather as reflecting changes in contrast and noise properties.

In addition, the Limitations section has been revised to state that the presented results rely on quantitative surrogate metrics and that observer-based validation remains an important topic for future investigation.
